# Anastomotic Repair versus Free Graft Urethroplasty for Bulbar Strictures: A Focus on the Impact on Sexual Function

**DOI:** 10.1155/2015/912438

**Published:** 2015-10-01

**Authors:** Matthias Beysens, Enzo Palminteri, Willem Oosterlinck, Anne-Françoise Spinoit, Piet Hoebeke, Philippe François, Karel Decaestecker, Nicolaas Lumen

**Affiliations:** ^1^Department of Urology, Ghent University Hospital, 9000 Ghent, Belgiumuzgent.be; ^2^Center for Urethral and Genital Surgery, 52100 Arezzo, Italy; ^3^Department of Urology, CH Mouscron, 7700 Mouscron, Belgium

## Abstract

*Objectives*. To evaluate alterations in sexual function and genital sensitivity after anastomotic repair (AR) and free graft urethroplasty (FGU) for bulbar urethral strictures. *Methods*. Patients treated with AR (*n* = 31) or FGU (*n* = 16) were prospectively evaluated before, 6 weeks and 6 months after urethroplasty. Evaluation included International Prostate Symptom Score (IPSS), 5-Item International Index of Erectile Function (IIEF-5), Ejaculation/Orgasm Score (EOS), and 3 questions on genital sensitivity. *Results*. At 6 weeks, there was a significant decline of IIEF-5 for AR (−4.8; *p* = 0.005), whereas there was no significant change for FGU (+0.9; *p* = 0.115). After 6 months, differences with baseline were not significant overall and among subgroups. At 6 weeks, there was a significant decline in EOS for AR (−1.4; *p* = 0.022). In the FGU group there was no significant change (+0.6; *p* = 0.12). Overall and among subgroups, EOS normalized at 6 months. After 6 weeks and 6 months, respectively, 62.2 and 52% of patients reported alterations in penile sensitivity with no significant differences among subgroups. *Conclusions*. AR is associated with a transient decline in erectile and ejaculatory function. This was not observed with FGU. Bulbar AR and FGU are likely to alter genital sensitivity.

## 1. Introduction

Although a short bulbar stricture can be treated by dilation or endoscopic urethrotomy, longer or recurrent strictures are best treated by urethroplasty as it provides the best chance of success [1–3]. Anastomotic repair (AR) and free graft urethroplasty (FGU) are established treatments for bulbar strictures with the choice of technique mainly depending on stricture length [1, 3, 4]. The main goal of urethroplasty is to restore urethral patency, and, as a consequence, most papers have focused on this criterion to evaluate success of urethroplasty [1, 3, 5]. In the past decade, there is an upcoming concern that especially bulbar urethroplasty might affect sexual functioning [6–8]. The aim of this paper is to evaluate and compare sexual function after AR and FGU for bulbar strictures in a prospective fashion.

## 2. Materials and Methods

### 2.1. Patient Recruitment

**Figure 1:**
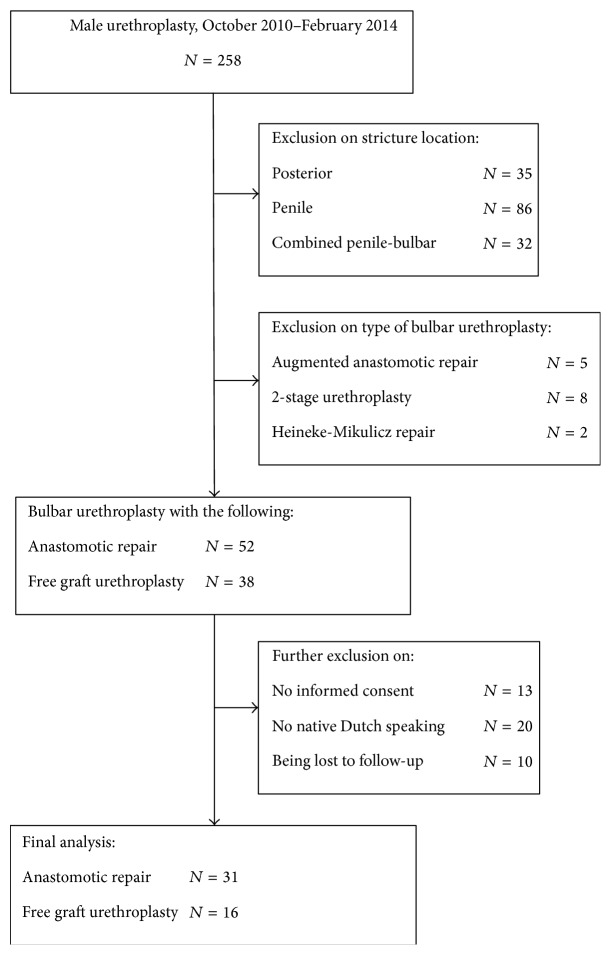
Flowchart of patient inclusion.

Out of 258 male patients who underwent urethroplasty between October 2010 and February 2014, 90 patients with a bulbar stricture only were planned to be treated with AR or FGU and eligible to participate in this prospective study. Only native Dutch speaking patients who signed the informed consent (Institutional Review Board Approval EC UZG 2008/234) and who filled in the preoperative questionnaires and at least one postoperative questionnaire (at 6 weeks and/or 6 months) were included in this analysis. Finally, 47 patients were included for further analysis and divided into two groups: AR (*n* = 31) versus FGU (*n* = 16) (Figure 1). Prepuce and oral mucosa was used as graft in, respectively, 12 and 4 patients. Stricture location and stricture length were evaluated by retrograde urethrography. This study included the following evaluations:

(i) urinary symptoms: maximum urinary flow (*Q*_max_) and the International Prostate Symptom Score (IPSS) questionnaire; the IPSS ranges from 0 (no lower urinary tract symptoms) to 35 (severe lower urinary tract symptoms);
(ii) erectile function: the abridged 5-item version of the International Index of Erectile Function (IIEF-5) [9]; this score ranges from 1 (no sexual intercourse) to 25 (no erectile dysfunction);
(iii) ejaculation/orgasm: the sum of questions 9 and 10 from IIEF (long version) [10]; this Ejaculation/Orgasm Score (EOS) ranges from 2 (no ejaculation/orgasm) to 10 (normal ejaculation and orgasm);
(iv) postoperative genital sensitivity: a nonvalidated in-house questionnaire containing 3 dichotomous questions on glans tumescence, alterations in genital sensitivity, and cold feeling in the glans; further analysis of glans tumescence was only done in patients reporting normal erectile function (IIEF-5 ≥ 20) in order to avoid contamination of diminished glans tumescence due to globally diminished penile tumescence.

Patients were evaluated preoperatively, after 6 weeks and 6 months. In the first six months, no phosphodiesterase-5 inhibitors were prescribed to stimulate sexual rehabilitation. In case of suspicion of stricture recurrence (*Q*_max_ < 15 mL/s and/or IPSS > 19), retrograde urethrography and urethroscopy were done. A functional definition of failure was used which includes the need for any additional urethral manipulation (including dilation) [11].

### 2.2. Surgical Technique

Patients were operated on in a single center (GUH) by two surgeons (Nicolaas Lumen and Willem Oosterlinck). AR was preferred whenever a tension-free anastomosis could be made (stricture length < 3 cm on urethrography and/or peroperative findings). For longer strictures, FGU was performed. For both techniques, a midline perineal incision is made; the bulbospongiosus muscle is incised at the midline and dissected away from the corpus spongiosum. In case of AR, the corpus spongiosum is circumferentially freed at the level of the stricture. The corpus spongiosum and urethra are transected at this site. The fibrotic urethra and spongiosus edges are resected until healthy urethra is present at both the distal and proximal ends. The urethra is then spatulated in order to obtain a broad oblique anastomosis, which is finalized by 8–10 interrupted resorbable 4.0 sutures. In case of FGU, the stricture is opened ventrally on the tip of the catheter. The stricture length is measured and a graft is taken accordingly. The graft is sutured into the urethra in a ventral onlay fashion. The corpus spongiosum is closed over the graft for vascular supply and mechanical support (spongioplasty). The urethral catheter is maintained for 14 days and a voiding cystourethrogram is made upon removal.

### 2.3. Statistical Analysis

Descriptive statistics were performed to evaluate the whole population and both subgroups. To compare both groups, continuous variables were evaluated by independent-samples *t*-test or the Welch modified *t*-test for, respectively, equal and unequal distributions. Categorical variables were evaluated by chi-square or Fischer's exact test. The 2-year recurrence-free survival was estimated by Kaplan-Meier statistics and groups were compared by log rank statistics. To evaluate changes in IPSS, IIEF-5 score, and EOS between baseline and at 6 weeks and 6 months, mean differences were calculated by paired-samples *t*-test.

## 3. Results

**Figure 2:**
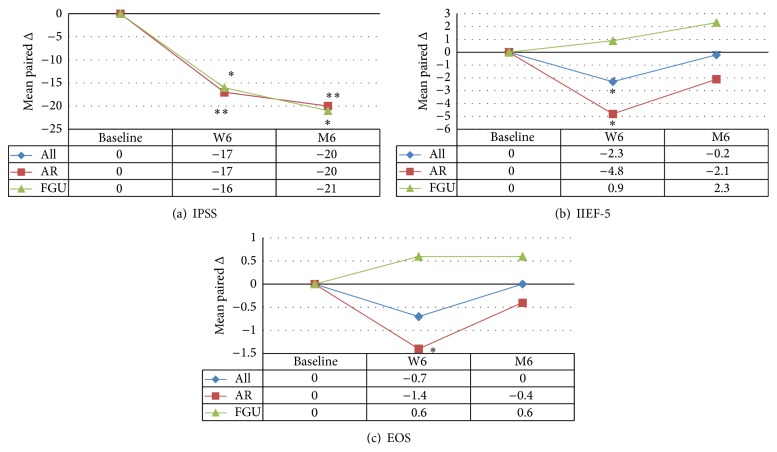
Evolution of International Prostate Symptom Score (a), International Index of Erectile Function-5 (b), and Ejaculation/Orgasm Score (c) for all patients and subdivided for anastomotic repair (AR) and free graft urethroplasty (FGU) (_ _^*∗*^ *p* < 0.05).

Patients treated by AR were significantly younger (37 versus 48 years; *p* = 0.018) and strictures were shorter with AR compared to FGU (1.8 versus 5.4 cm; *p* < 0.001). Both groups were comparable for follow-up duration, stricture etiology, previous interventions, and presence of suprapubic catheter and for preoperative urinary flow, IPSS, IIEF-5, and EOS (Table 1). After a mean follow-up of 23 months, 6 patients (12.8%) suffered a recurrence: 3 (9.7%) patients treated with AR and 3 (18.8%) patients treated with FGU (*p* = 0.395). Estimated 2-year recurrence-free survival rate was 93% and 72%, respectively, for AR and FGU (*p* = 0.347). Overall and in both groups, there was a significant improvement of the urinary flow at latest follow-up. Accordingly, there was a significant improvement in IPSS after 6 weeks and 6 months overall and in both groups (Table 2; Figure 2(a)).

Thirty-three patients, respectively, 19 and 14 patients in the AR- and FGU-group, reported to have sexual intercourse and filled in the IIEF-5 (Table 3; Figure 2(b)). Overall, there was a significant decline in IIEF-5 score after 6 weeks (−2.3; *p* = 0.026). This decline remained significant for AR (−4.8; *p* = 0.005). However, for FGU, there was no significant change in IIEF-5 score (+0.9; *p* = 0.115). After 6 months, there were no longer significant changes in IIEF-5 score overall (−0.2; *p* = 0.907), for AR (−2.1; *p* = 0.263) and for FGU (+2.3; *p* = 0.313).

Thirty-seven patients, respectively, 23 and 14 patients in the AR- and FGU-group, tried to have ejaculation/orgasm (by masturbation or sexual intercourse) and completed the EOS (Table 3; Figure 2(c)). Overall, there was no significant postoperative change in EOS at 6 weeks (−0.7; *p* = 0.111). However, in the AR-group there was a significant decline in EOS (−1.4; *p* = 0.022). This was not the case in the FGU-group (+0.6; *p* = 0.12). After 6 months, EOS returned to baseline. The decline for AR (−0.4; *p* = 0.431) was no longer significant.

At 6 weeks and 6 months, respectively, 45 and 25 patients filled in the questionnaire on genital sensitivity and on cold feeling in the glans. At 6 weeks, 28 patients (62.2%) reported to have altered genital sensitivity. This proportion was not significantly different between AR and FGU (66.7 versus 53.3%; *p* = 0.517). Only one patient, treated by AR, had a cold feeling in the glans. At 6 months, 13 patients (52%) reported to have altered genital sensitivity. Again, this proportion was not significantly different with AR compared to FGU (58.8% versus 37.5%; *p* = 0.411). At 6 months, no one reported a cold feeling in the glans. Of 20 patients with IIEF-5 ≥ 20 at 6 weeks 1/10 (10%) and 4/10 (40%) of patients in, respectively, the AR- and FGU-groups reported no glans tumescence (*p* = 0.303). At 6 months, 1/6 (16.7%) and 3/5 (60%) patients with IIEF-5 ≥ 20, respectively, treated by AR and FGU reported no glans tumescence (*p* = 0.242). Of the 4 patients treated with oral mucosa, 2 had altered genital sensitivity and no glans tumescence at 6 weeks and 6 months.

## 4. Discussion

Although this series is a prospective study, no randomization was done between AR and FGU because the use of AR is limited by the stricture length. The limit for AR is usually set at 2-3 cm [4, 12]. This also explains why strictures treated with AR were significantly shorter compared to FGU in this series. Another difference between both groups was younger patient's age with AR. For this observation, we have the following explanation: patients treated with AR have shorter strictures (cf. supra) and short bulbar strictures are predominantly idiopathic/congenital in origin and thus occurring at a younger age [13]. Despite these differences in age and stricture length between AR and FGU, preoperative erectile and orgasmic function was not significantly different between these groups. It has been reported that longer stricture length and more advanced patient age are more likely to be associated with postoperative erectile dysfunction (ED) [14–16]. The observed difference in patient age and stricture length would thus be in favor of AR in terms of postoperative erectile function. This has not been observed in this series, on the contrary.

The success rate of 90.3% for AR in this series is in line with the 93.8% composite success rate reported by the SIU/ICUD consultation [1]. For longer strictures at the bulbar urethra, FGU is the preferred technique of substitution urethroplasty as flaps are associated with more morbidity [3]. Our 81.2% success rate of ventral FGU is again in line with the overall 88.8% success rate reported by the SIU/ICUD consultation [3]. Because of its excellent success rate, the SIU/ICUD consultation recommends AR as optimal treatment for short bulbar strictures [1]. This recommendation is questioned because of a potential higher risk of sexual dysfunction related to AR [17].

An increasing number of papers report on sexual dysfunction after urethroplasty [6–8, 18]. Although the results are far from uniform, there is a trend for a higher incidence of sexual dysfunction after AR compared to FGU. Palminteri et al. found that 35% and 65% of patients treated by FGU reported improvement in erectile and ejaculatory function [8]. This is in line with our results revealing a trend to improvement in erectile and orgasmic function in the FGU-group. Al-Qudah and Santucci reported ED as late complication in 17% of patients after AR but no ED after FGU [18]. In their prospective study, Erickson et al. found the highest incidence of ED (50%) in the group treated by AR, compared to FGU, where only 26% of patients suffered from ED. However, these differences were not statistically significant [7]. In their logistic regression model, Xie et al. reported that the method of treatment is a significant factor to predict for postoperative ED, with the highest risk of ED for AR [6].

Other authors did not find a significant decline in erectile function [19, 20] nor did they find a difference between AR and FGU [15, 16, 21, 22]. These contradictory results can be explained by several factors. First, timing of evaluation seems to be very important. Erickson et al. found a significant worse erectile function when evaluation is done <1 year after urethroplasty [15]. Xie et al. found a significant decline of erectile function with AR after 3 months but a normalization after 6 months [6]. This was also noted by Mundy, who found ED in 53% and 33% of patients after AR and FGU, respectively, at a 3-month follow-up. This decreased to 5% and 0.9% after longer follow-up [23]. In the AR-group, we also found a transient decline in erectile function after 6 weeks with recuperation after 6 months. Therefore, it is likely that if erectile function is at earliest assessed >3 months after urethroplasty [19, 22], a transient decline in erectile function might have been missed. Secondly, the evaluation tool to assess erectile function might be important. The IIEF-5 is a validated questionnaire to assess erectile function and was therefore used in this series. Other authors, however, used an in-house questionnaire with dichotomous answers (erectile dysfunction present or absent) [16, 19, 22]. Other factors that might be important to explain contradictory findings among studies are retrospective evaluation (with risk of recall bias) [9, 16, 19, 22] and small patient groups [21].

**Figure 3:**
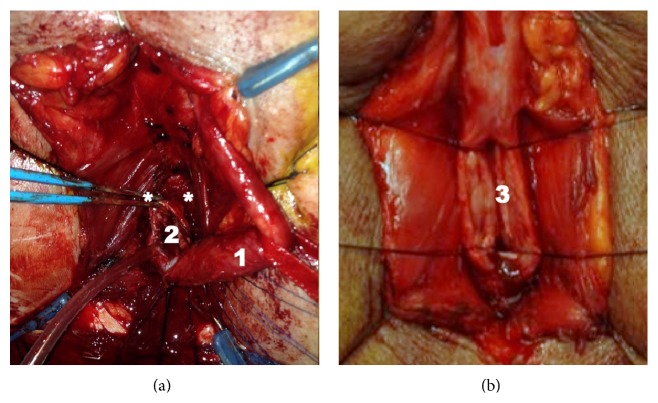
Peroperative photographs of AR (a) and FGU (b): a more extensive dissection with AR can be appreciated; 1: circumferentially mobilized bulbar urethra; 2: transected urethra; *∗*: region where erectile nerves are expected; and 3: ventrally opened bulbar urethra.

We speculate that the observed transient decline in erectile function with AR might be related to the following:

- more extensive and circumferential dissection of the corpus spongiosum containing the bulbar urethra; proximal dissection and mobilization of the corpus spongiosum nearby the urogenital diaphragm and in the intracrural space might provoke neuropraxia and/or thermal damage (coagulation) of erectile nerves penetrating the corporal bodies at that location (Figure 3); this hypothesis is supported by neuroanatomical findings reported by Yucel and Baskin [24] and Akman et al. [25];
- complete transection of the corpus spongiosum that might be associated with a higher risk of bleeding and with postoperative haematoma and inflammation; this needs some time to recover; this might withhold patients to have satisfactory sexual activity or might provoke psychological problems.

In this series, ventral FGU was performed, with no significant decrease in sexual functioning at 6 weeks and 6 months. It would be interesting to know whether dorsal FGU affects sexual functioning. One would expect a higher incidence of sexual dysfunction if the hypothesis of more extensive and circumferential dissection of the bulbar corpus spongiosum is (in part) responsible for sexual dysfunction.

In this series, a transient decline in EOS was seen with AR, whereas there was no significant difference observed with FGU. Erickson et al. found an improvement of ejaculatory function after urethroplasty (mix of AR and FGU) [15], but a later prospective study failed to show any significant changes in ejaculatory function after urethroplasty (also mix of AR and FGU) [26]. Improvement of ejaculatory function after urethroplasty might be related to desobstruction of the urethra [26]. However this cannot explain the transient decline in ejaculatory function after AR that was seen in our series. Barbagli et al. also reported postoperative ejaculatory dysfunction in 23.3% of patients treated with AR [19]. We hypothesize that the higher rate of ejaculatory dysfunction associated with AR is because of the more extensive detachment of the bulbospongiosus muscle in AR needed for a full mobilization of the bulbar urethra. This detachment can indeed interfere with ejaculatory function. Timing of questioning might again be important: recovery of postoperative ejaculatory dysfunction can be expected once the bulbospongiosus muscle has recovered from the surgical trauma. This cannot be expected after 6 weeks but can be expected after 6 months. Another explanation is that ejaculatory and orgasmic dysfunction is related to ED, which was also more frequent after AR.

In this series, postoperative changes in genital sensitivity were present in approximately 2 out of 3 and 1 out of 2 patients after, respectively, 6 weeks and 6 months. Changes in genital sensitivity were not significantly different among subgroups. Palminteri et al. found a change in genital sensitivity after FGU in 50% of patients [8]. This is in line with our findings, but substantially higher than the 18.3% reported rate by Barbagli et al. [19]. However, this was a retrospective series with a possible risk of underreporting. In the same series [19], only one patient (1.6%) reported a cold glans, which is in concordance with the finding in our series. Postoperative changes in genital sensitivity might be explained by postoperative haematoma formation, oedema, and inflammation. Furthermore, in the majority of patients treated by FGU, a preputial skin graft was used. These factors might certainly explain the high rate of early (6 weeks) changes in genital sensitivity. However, even after 6 months, changes in genital sensitivity were still frequently reported, and this occurs also in patients treated with oral mucosa. This might be explained by damage to some sensory branches of the perineal nerves that supply the ventral surface of the penis [24]. By transecting the entire corpus spongiosum, one would expect a higher rate of impaired glans tumescence after AR. This was not observed in this series. However, interpretation of the results is hampered by the small number of patients.

This series again underlines the concern of possible alterations in sexual functioning and genital sensitivity after bulbar urethroplasty. Therefore it should be part of the evaluation of patients treated by urethroplasty. Jackson et al. recently validated patient reported outcome measures (PROMs) for urethroplasty [27]. However, this PROM lacks a section on sexual functioning.

Furthermore, it would be interesting to evaluate whether modifications in urethroplasty techniques such as muscle- and nerve-sparing bulbar urethroplasty [28] and vessel-sparing anastomotic repair [29] will be associated with less sexual dysfunction.

Important limitations of the present series are the small sample size and the missing data in the postoperative questionnaires.

## 5. Conclusions

AR is associated with a transient decline in erectile and ejaculatory function. This was not observed with FGU. Bulbar urethroplasty is likely to provoke changes in genital sensitivity. Further prospective studies with validated and internationally accepted patient reported outcome measures (PROMs) are needed for further confirmation.

## Figures and Tables

**Table 1:** Patients' characteristics (SD = standard deviation; FGU = free graft urethroplasty; AR = anastomotic repair; DVIU = direct vision internal urethrotomy; *Q*_max⁡_ = maximum urinary flow; IPSS = International Prostate Symptom Score; IIEF = International Index of Erectile Function; EOS = Ejaculation/Orgasm Score).

		All (*n* = 47)	FGU (*n* = 16)	AR (*n* = 31)	*p* value
Age (years)	Mean (SD)	40 (16)	48 (18)	37 (13)	*0.018 *
Follow-up (months)	Mean (SD)	23.3 (10.9)	25.2 (12.5)	22.2 (10)	0.376
Stricture length (cm)	Mean (SD)	3 (2.4)	5.4 (2.6)	1.8 (0.8)	*<0.001 *
Stricture etiology					
Traumatic	Number (%)	4 (8.5)	0 (0)	4 (12.9)	0.071
Inflammatory	Number (%)	1 (2.1)	0 (0)	1 (3.2)	
Iatrogenic	Number (%)	14 (29.8)	8 (50)	6 (19.4)	
Idiopathic	Number (%)	28 (59.6)	8 (50)	20 (64.5)	
Previous interventions					
None	Number (%)	4 (8.5)	2 (12.5)	2 (6.5)	0.877
DVIU/dilation(s)	Number (%)	34 (72.3)	11 (68.8)	23 (74.2)	
Urethroplasty(ies)	Number (%)	9 (19.1)	3 (18.8)	6 (19.4)	
Preop *Q*_max⁡_ (mL/s)	Mean (SD)	6.3 (4.6)	6.9 (4)	6 (5)	0.629
Preop IPSS (…/35)	Mean (SD)	22 (8)	23 (7)	21 (8)	0.368
Preop IIEF-5 (…/25)	Mean (SD)	20 (7)	18 (8)	22 (6)	0.202
Preop EOS (…/10)	Mean (SD)	8 (3)	7 (4)	9 (3)	0.135
Suprapubic catheter					
Yes	Number (%)	8 (17)	2 (12.5)	6 (19.4)	0.697
No	Number (%)	39 (83)	14 (87.5)	25 (80.6)	

**Table 2:** Mean paired differences (Δ) of the maximum urinary flow (*Q*_max⁡_) and International Prostate Symptom Score (IPSS). The standard deviation is provided between brackets (FGU = free graft urethroplasty; AR = anastomotic repair).

	Δ*Q*_max⁡_ (mL/s)	*p* value	ΔIPSS (6 weeks versus preop)	*p* value	ΔIPSS (6 months versus preop)	*p* value
All	+19.8 (13.9)	*<0.001 *	−17 (8)	*<0.001 *	−20 (9)	*<0.001 *
FGU	+13.8 (11.7)	*0.007 *	−16 (10)	*<0.001 *	−21 (8)	*<0.001 *
AR	+22.3 (14.3)	*<0.001 *	−17 (7)	*<0.001 *	−20 (9)	*<0.001 *

**Table 3:** Mean paired differences (Δ) of the 5-Item International Index of Erectile Function (IIEF-5) and Ejaculation/Orgasm Score (EOS). The standard deviation is provided between brackets (FGU = free graft urethroplasty; AR = anastomotic repair).

		ΔIIEF-5 (6 weeks versus preop)	*p* value		ΔIIEF-5 (6 months versus preop)	*p* value
All	*n* = 33	−2.3 (5.8)	*0.026 *	*n* = 18	−0.2 (6)	0.907
FGU	*n* = 14	+0.9 (2)	0.115	*n* = 8	+2.3 (5.8)	0.313
AR	*n* = 19	−4.8 (6.5)	*0.005 *	*n* = 10	−2.1 (5.6)	0.263

		ΔEOS (6 weeks versus preop)	*p* value		ΔEOS (6 months versus preop)	*p* value

All	*n* = 37	−0.7 (2.5)	0.111	*n* = 22	0 (1.9)	1
FGU	*n* = 14	+0.6 (1.3)	0.12	*n* = 8	+0.6 (2.2)	0.448
AR	*n* = 23	−1.4 (2.8)	*0.022 *	*n* = 14	−0.4 (1.6)	0.431
